# Chronic pain after lower abdominal surgery: do *catechol-O-methyl transferase/opioid receptor μ-1* polymorphisms contribute?

**DOI:** 10.1186/1744-8069-9-19

**Published:** 2013-04-08

**Authors:** Yuri Kolesnikov, Boris Gabovits, Ariel Levin, Andres Veske, Li Qin, Feng Dai, Inna Belfer

**Affiliations:** 1 East Tallinn Central Hospital, Tallinn Technological University, Tallinn, Estonia; 2 Department of Gene Technology, Tallinn Technological University, Tallinn, Estonia; 3 Yale Center for Analytical Sciences, New Haven, CT, USA; 4 Departments of Anesthesiology & Human Genetics, University of Pittsburgh, Pittsburgh, PA, USA

**Keywords:** Chronic postsurgical pain, Lower abdominal surgery, COMT, OPRM1, Gene, Polymorphism

## Abstract

**Background:**

Preoperative pain, type of operation and anesthesia, severity of acute postoperative pain, and psychosocial factors have been identified as risk factors for chronic postsurgical pain (CPP). Recently, it has been suggested that genetic factors also contribute to CPP. In this study, we aimed to determine whether the catechol-O-methyl transferase (*COMT*) and opioid receptor μ-1 (*OPRM1*) common functional polymorphisms rs4680 and rs1799971 were associated with the incidence, intensity, or duration of CPP in patients after lower abdominal surgery.

**Methods:**

One hundred and two patients with American Society of Anesthesiologists (ASA) physical status I/II underwent either abdominal radical prostatectomy (n = 45) or hysterectomy (n = 57). The incidences of CPP in the pelvic and scar areas were evaluated in all patients three months after surgery.

**Results:**

Thirty-five (34.3%) patients experienced CPP after lower abdominal surgery. Within this group, six (17.1%) patients demonstrated symptoms of neuropathic pain. For *COMT* rs4680, 22 (21.6%) patients had Met158Met, 55 (53.9%) patients had Val158Met, and 25 (24.5%) patients had Val158Val. No association was found between CPP phenotypes (incidence, intensity, and duration) and different rs4680 genotypes. For *OPRM1* rs1799971, only CPP patients carrying at least one copy of the G allele had higher pain intensity than A118A carriers (p=0.02). No associations with other phenotypes were found. No combined effect of *COMT/OPRM1* polymorphisms on CPP phenotypes was observed.

**Conclusions:**

*OPRM1* genotype influences CPP following lower abdominal surgery. *COMT* didn’t affect CPP, suggesting its potential modality-specific effects on human pain.

## Background

Chronic postsurgical pain (CPP) varies between type of operation and between patient populations, but it is clearly a common complication after surgery [1]. Surgery itself or tissue damage pose a significant risk for chronic or long-lasting pain; however, the variability in this pain cannot be explained only by surgery- or treatment-related factors [2]. Thus, the identification of other risk factors for CPP is important for the prediction of patients at risk and development of strategies to prevent CPP. Pre-operative pain, type of operation and anesthesia, postoperative pain severity, and psychosocial characteristics have been identified as risk factors for this complication [1,3]. Recently, it has been suggested that genetic factors also contribute to CPP [4,5]. Several genetic polymorphisms were found to be associated with different types of chronic pain, such as in the GTP cyclohydrolase enzyme (*GCH1*) [6], potassium channel subunit (*KCNS1*) [7], and catechol-O-methyl transferase (*COMT*) [8] genes, which play a role in persistent radiculapathic pain following surgical discectomy [6,7], postamputation pain [7], and chronic orafacial pain [8], respectively. *COMT* contains the well-known common functional single nucleotide polymorphism (SNP) rs4680, or *G1947A,* encoding for *Val158Met* substitution. Homozygosity for the *158Met* allele is associated with a three- to four-fold reduction of *COMT* enzyme activity, compared to homozygosity for the *158Val* variant, resulting in reduced degradation of synaptic catecholamines [9]. Zubieta and colleagues found that *COMT Val158Met* polymorphism contributed to pain phenotypes via μ-opioid-related mechanisms [10]. Homozygosity for the *158Met* allele was associated with diminished regional μ-opioid system responses to pain and increased μ-opioid receptor binding potential [10]. Thus, an increase in the density of μ-opioid receptors possessing a homozygous *Met158Met* genotype may result in the improved efficacy of morphine as well as endogenous opioids during stress. The gene encoding opioid receptor μ-1 (*OPRM1)* also has a well-established functional SNP rs1799971, or *A118G,* that contributes to human pain [11]. Recently, we published a study where we investigated the joint effect of *COMT* and *OPRM1* polymorphisms on the morphine response in postoperative analgesia [12]. We found that in patients undergoing lower abdominal surgery, the first 48 hours of morphine consumption and acute pain relief varied according to genotype. Joint carriers of *OPRM1, A118G,* and *COMT Val158Met* required a morphine dosage 18% less than that of *OPRM1, A118A,* and *COMT Val158Val* carriers [12]. In the present study, we used the same patient population to determine whether *COMT* and *OPRM1* polymorphisms contribute to chronic pain after lower abdominal surgery.

## Results

We studied 102 patients 34.3% (n = 35) who experienced postsurgical pain in the pelvic and scar regions three months after lower abdominal surgery. The average intensity of pain was 3.7±1.5 (pain score on a Numeric Rating Scale [NRS]), and the average duration was 10.3±12.0 days. Within this group, six patients (17.1%) demonstrated symptoms of neuropathic-like pain at pain clinic examination. Twenty of 35 CPP patients used pain medications including diclofenac, paracetamol, and/or ibuprofen to alleviate pain.

We found no statistically significant difference in patient demographics between the CPP and non-CPP groups: the mean ages of each group were 53.9±9.8 and 54.8±10.6, respectively (p=0.65). The gender distributions (40.0% male vs. 46.3% female) were also similar (p=0.55). There were no significant differences in intensity of acute pain or morphine consumption in the early postoperative period (first 48 hours) between patients with CPP and patients without it (p = 0.13). Furthermore, we found no statistical differences in the intensity of acute pain and CPP between males and females (Additional file 1: Table S1) or in the distribution patterns of either *OPRM1, A118G, or COMT G1947A* SNP between males and females in all of the patients, the CPP patients, and the non-CPP patients, respectively (Additional file 2: Table S2).

Table 1 displays allelic frequency data for *COMT* rs4680 and *OPRM1* rs1799971*.* For *COMT* rs4680, 22 (21.6%), 55 (53.9%), and 25 (24.5%) patients had the *A1947A (Met158Met), G1947A (Val158Met),* and *G1947G (Val158Val)* genotypes, respectively. For *OPRM1* rs1799971, 3 (2.9%), 15 (14.7%), and 84 (82.4%) patients had the *G118G, A118G, and A118A* genotypes, respectively. Both SNPs were in Hardy-Weinberg Equilibrium (Table 1).

**Table 1:** **Summary of information for *COMT *rs4680 *or OPRM1 *rs1799971**

			**Alleles**^**a**^			**Genotype**			
SNP	Chr	Position	A1	A2	MAF^b^	A1/A1	A1/A2	A2/A2	HWE^c^
rs4680	22	19951271	A (Met)	G (Val)	0.49	22 (21.6%)	55 (53.9%)	25 (24.5%)	0.55
rs1799971	6	154402490	G	A	0.10	3 (2.9%)	15 (14.7%)	84 (82.4%)	0.07

^a^: A1: Minor or Variant allele; A2: Major or wild-type allele.

^b^: MAF: Minor allelic frequency.

^c^: Hardy-Weinberg Equilibrium (HWE) test *P*-value.

Our analyses of the 102 patients did not show a significant association between the two SNPs and the incidence of CPP (rs4680, *P* = 0.74; rs1799971, *P* = 0.31) under the additive genetic effect model after adjustment for baseline pain score (postoperative acute pain in the post-anesthesia care unit [PACU]), age, and gender. No significant associations were identified assuming the dominant or recessive genetic model of variant or minor allele of the two SNPs (Table 2).

**Table 2:** **Association of *COMT *rs4680 *or OPRM1 *rs1799971 genotypes with CPP incidence in 102 patients**

**SNP**	**Genotypes**	**CPP**	**No CPP**	**Total**	***P***_***additive***_	***P***_***dominant***_	***P***_***recessive***_
rs4680	Met158Met (AA)	10	12	22 (21.6%)			
	Val158Met (GA)	16	39	55 (53.9%)			
	Val158Val (GG)	9	16	25 (24.5%)	0.74	0.44	0.79
rs1799971	A118A	31	53	84 (82.4%)			
	A118G	3	12	15 (14.7%)			
	G118G	1	2	3 (2.9%)	0.31	0.22	0.95

*P*_additive_, *P*_dominant,_*P*_recessive_: *P*-value from logistic regression analysis of CPP status, assessing the additive, dominant and recessive genetic effect of rs4680 (or rs1799971) after adjusting for baseline pain score, age and gender.

In the CPP group (35 patients), our analyses of rs4680 did not identify any significant association with the intensity of pain after adjusting for baseline acute pain, age, and gender, assessing the additive (*P* = 0.30), dominant (*P* = 0.27), and recessive (*P* = 0.57) genetic models of the variant or minor allele (Table 3). There was also no association of rs4680 with duration of pain under the three genetic effect models. For rs1799971, only one CPP patient had the G118G genotype; thus, the recessive genetic model could not be assessed. We observed a gene-dosage (worsening) effect of the rs1799971 variant allele G; that is, carriers of at least one copy of the G allele had higher pain intensity scores than homozygous A118A carriers (*P* = 0.02, adjusted for all covariates). No statistical difference was found in duration of pain among CPP patients with different genotypes of rs1799971 (Table 3).

**Table 3:** **Association of *COMT *rs4680 *or OPRM1 *rs1799971 genotypes with pain score or duration of pain (days) in 35 CPP patients**

	***COMT *rs4680**			***OPRM1 *rs1799971**			***P-value (COMT, OPRM1)***		
	Val158Val (GG; n = 10)	Val158Met (GA; n= 17)	Met158Met (AA; n = 8)	A118A (n = 31)	A118G (n = 3)	G118G (n = 1)	Additive	Dominant	Recessive
Pain Score	3.8 ± 1.2	4.0 ± 1.5	3.3 ± 1.7	3.5 ± 1.3	5.3 ± 2.3	6	0.30, 0.02	0.27, 0.02	0.57, NA
Duration	11.1 ± 10.5	11.7 ± 15.3	6.5 ± 2.3	10.3 ± 12.1	13.0 ± 14.9	3	0.44, 0.37	0.48, 0.60	0.57, NA

Data are expressed as mean ± SD.

*P*-value was from the ANCOVA of CPP score (or duration of pain) after adjusting for pain medication, age, sex, and baseline pain score, assessing additive, dominant, and recessive genetic model of two SNPs. Recessive effect of G allele for rs179991 was not assessed as there was only patient with G118G.

We identified four common (frequency > 5%) combined *COMT/OPRM1* genotypes: Val158Val (GG) /A118A (21.6%), Met158Val (GA) /A118A (43.1%), Met158Met (AA)/A118A (17.6%), and Met158Val (GA) /A118G (8.8%) (Table 4). No significant associations of these genotypes with the incidence of CPP were found (p = 0.46). However, among the CPP patients, carriers (N = 2) of Met158Val (GA) /A118G had higher pain intensity scores than carriers with the other three common combined genotypes (p =0.005-0.029, Table 5). No significant differences in pain duration were found among CPP patients with different *COMT/OPRM1* genotypes.

**Table 4:** **Association of combined *COMT/OPRM1 *genotypes with incidence of CPP in 102 patients**

**Combined *COMT/OPRM1 *genotypes**	**CPP**	**No CPP**	**Total**
Val158Val (GG)/A118A	10	12	22 (21.6%)
Met158Val (GA)/A118A	14	30	44 (43.1%)
Met158Met (AA)/A118A	7	11	18 (17.6%)
Met158Val (GA) /A118G	2	7	9 (8.8%)
Met158Met (AA) /A118G	1	3	4 (3.9%)
Met158Val (GA) /G118G	1	1	2 (2.0%)
Val158Val (GG) /A118G	0	2	2 (2.0%)
Val158Val (GG) /G118G	0	1	1 (1.0%)
Met158Met (AA) /A118G	0	0	0 (0%)
Total	35	67	102

Note: Association of common combined genotypes (freq > 5%, first four rows) with the incidence of CPP was tested using a logistic regression analysis, after adjusting for age, gender and baseline pain score (*P*-value = 0.46).

**Table 5:** **Association of combined *COMT/OPRM1 *genotypes with pain score in CPP patients**

		**Combined genotypes *COMT/OPRM1***			
		**Met158Val (GA)/A118G (n = 2)**	**Val158Val (GG)/A118A (n = 10)**	**Met158Val (GA)/A118A (n = 14)**	**Met158Met (AA)/A118A (n = 7)**
Pain Score	Adjusted mean (95% CI)^*^	6.4 (4.6, 8.2)	3.4 (2.5, 4.2)	3.3 (2.6, 4.0)	4.0 (3.1, 5.0)
	Difference (95% CI), *P*-value	Reference	−3.0 (−5.1, -1.0), 0.005	−3.1 (−5.1, -1.0), 0.005	−2.4 (−4.5, -0.3), 0.029
Duration of Pain	Adjusted mean (95% CI)^*^	12.1 (−6.5, 30.7)	15.4 (6.8, 24.0)	14.3 (7.0, 21.6)	10.3 (0.2, 20.4)
	Difference (95% CI), *P*-value	Reference	3.3 (−17.2, 23.9), 0.74	2.2 (−18.7, 23.1), 0.83	−1.7 (−23.3, 19.8), 0.87

^*^: Adjusted least square means after adjusting for baseline pain, pain medication, score age, gender. No significant difference in either pain.

score, or duration of pain among Val158Val (GG)/A118A, Val158Val (GG)/A118A, and Val158Val (GG)/A118A.

## Discussion

Chronic postsurgical pain is a prevalent, though often overlooked healthcare problem. Twenty-five percent of patients attending pain clinics reported surgery as the cause of chronic pain, with an estimated CPP incidence of 10-65% [13,14]. The incidence of chronic neuropathic pain one year after surgery is between 0.5 and 1.5% [15,16]. Thirty-five (34.3%) patients in our study had residual persistent pain three months after lower abdominal surgery, and six patients had neuropathic pain syndrome. These findings are consistent with previous studies that reported pain incidences of up to 32% after lower abdominal surgery in long-term follow up [14]. The mechanisms of CPP are complex and not fully understood. Even for a single surgical procedure, different pain syndromes have different mechanisms of pathology [17]. One of the direct causes of postoperative chronic pain is scar formation. Scar tissue forms when the skin and tissue begin to heal, often leading to the pulling of surrounding tissues, compression or irritation of nerve endings, and the entrapment of nerve cells within the scar tissue itself [17,18]. In addition, surgery can cause the release of inflammatory mediators that activate primary afferent nociceptors, and persistent pain can lead to peripheral sensitization. If persistent pain ceases in the process of normal wound healing, sensitization and facilitation of synaptic transmission to the central nervous system (CNS) is reversed and normal nociceptor activity is restored [19,20]. The after-effects of surgery, such as prolonged inflammation due to the insertion of mesh materials or chronic nerve stretching, can lead to sensitization and facilitation of synaptic transmission that subsequently cause phenotypic and pathophysiological changes in nociceptors [21]. These include changes in gene expression, receptor translocation to the cell membrane, sustained activation of glial cells, and spinal facilitation of nociception. These structural changes lead to pain amplification, later resulting in CPP and discomfort around the surgical area [17].

Nonetheless, not all patients experience pain around the scar area after surgery. Genetics, as well as interaction between genetic and environmental factors, are likely to influence inter-individual variability in CPP [5,9]. Namely, functional genetic polymorphisms of the *COMT* gene are associated with altered sensitivity to pain induced in an experimental (controlled) environment. Low *COMT* activity poses a high risk for the development of chronic orafacial pain and fibromyalgia [22]. Decreased activity of the *COMT* enzyme encoded by the *158Met* allele (variant allele of rs4680) may result in accumulation of epinephrine and norepinephrine in the peripheral and CNS, leading to overstimulation of nociceptive beta 2/3-adrenergic pathways and high pain sensitivity as observed in *118G* carriers [23]. Furthermore, *COMT* inhibition causes increased pain sensitivity comparable to the pro-nociceptive effects of carrageenan mediated by beta 2/3-adrenergic mechanisms [23]. Recently, we showed the contribution of *COMT* functional variation to clinical outcome after surgical treatment for disk degenerative disease [24]. We also showed that cumulative pain scores at rest recorded postoperatively in the same cohort of surgery patients as used in this study were lower in the first two postoperative days in the *COMT* rs4680 Val/Val homozygous group compared to the Met/Met homozygous group [12]. In this study, we report no statistically significant association between *COMT* rs4680 and the incidence, intensity, or duration of CPP. These findings suggest the modality-specific nature of *COMT* effects, e.g. it contributes to acute postoperative pain after lower abdominal surgery, but not persistently or chronically. These data are important, since *COMT*’s effects on human pain are still under investigation, and more evidence is needed for a complete picture. That *COMT* is not associated with several pain conditions such as neuropathic pain syndromes already points to possible modality-specific effects [25]; this study provides further evidence. Since *COMT* genotyping may potentially have value for clinical decision-making based on predicted pain severity or time course or patients’ response to analgesia [26], it is essential to further evaluate *COMT*’s effects in different pain models and for multiple pain phenotypes to identify precisely the array of clinical settings in which *COMT* could be used as a “pain biomarker”. Although our results provide additional knowledge on the relationship between *COMT* and CPP, they are limited to only one time point (three months postoperatively) and should be confirmed in a larger clinical setting using protocols specifically designed to investigate the intensity, quality, and duration of pain in scar regions at different time points after surgery.

Functional genetic polymorphisms between altered pain thresholds and analgesic responses to opioid administration for the *OPRM1* A118G genotype (rs1799971) have been well characterized [11]. Previously, we evaluated these alleles in an abdominal surgery patient cohort (this study cohort) in relation to acute postoperative pain, opioid consumption, and side effects. We found that patients with A allele(s) had significantly less nausea scores compared to the G118 homozygotes, but no association was observed with pain itself [12]. In the current study, no association was found between this *OPRM1* SNP and the incidence of CPP; however, the intensity of reported pain was significantly higher in carriers of the G allele compared to the homozygous AA carriers. Interestingly, these data match results from several studies in other patient populations where the G allele was found to be associated with elevated mechanical pain responses, [12] as well as reduced response to morphine and other opioids in patients receiving treatment for postoperative or chronic pain [27,28]. A larger-scale study is therefore needed to confirm that the *OPRM1* G allele is a risk factor for chronic postsurgical pain and elucidate *OPRM1*’s effects on other human pain models.

Although no significant association was found between combined *COMT/OPRM1* genotypes and the incidence of chronic postsurgical pain in our study population, the CPP patients with Met158Met/A118G had higher pain scores than those patients with Val158Val/A118A, Met158Val/A118A, and Met158Met/A118A. However, as the sample size was too small to perform any powerful analyses, the results need to be interpreted very cautiously and more studies with larger sample sizes are needed to validate this finding.

It is important to acknowledge a few limitations in our study. Both genes tested have complex genetic structures, and the two SNPs tested do not cover the diversity of *COMT* and *OPRM1* variation [5]. Although these SNPs are the most studied and have proven functional consequences, it is possible that other functional polymorphisms in these genes affect pain phenotypes related to CPP. This study sample size didn’t allow us to assess other functional but less common alleles. Furthermore, we had limited statistical power to look for gene by gene interaction effects of CPP, and didn’t evaluate psychological factors that may shape the effects of these genes.

## Conclusion

No statistically significant association was found between the incidence of CPP after lower abdominal surgery and *COMT/OPRM1* polymorphisms in this patient population. Nevertheless, this data further improves our understanding of the genetic background of different types of CPP, and of the complexity of the effects of *COMT* and *OPRM1* polymorphisms on human pain.

## Methods

This study was approved by the local Human Studies Committee in Tallinn, Estonia (No. 1356; 17.04.2008). After providing informed consent, 102 Caucasian patients with ASA physical status I/II (57 women and 45 men) underwent abdominal surgery (for abdominal radical hysterectomy or prostatectomy, respectively [12]). Baseline measures of arterial blood pressure, heart rate, respiratory rate, and oxygen saturation were obtained by pulse oximetry before initiation of anesthesia. Before anesthesia and surgery, 5 mL of blood was collected from each patient and stored for genetic analysis.

### Intraoperative anesthesia

A standardized, general anesthesia technique was used for all patients [12]. For induction of anesthesia, fentanyl (2 mcg/kg), propofol (2 mg/kg), and atracurium (0.3 mg/kg) were used. After induction, inhaled sevoflurane and atracurium were chosen to maintain anesthesia. At the end of the surgery, residual neuromuscular block was antagonized with 2.5 mg of neostigmine and 1 mg of atropine; patients were tracheally intubated and transferred to the PACU.

*Postoperative analgesia:* After arrival at the PACU, patients were asked every 30 minutes whether they needed analgesics until they were alert enough to use the patient-controlled analgesia (PCA) pump [12]. When analgesics were required, 0.02 mg/kg of IV morphine was administered. The pump was set to deliver at 1 mg/h (background infusion), after which a 1 mg bolus dose with a lockout time of 10 minutes was given. In cases where the maximum permitted dose of morphine was reached and the patient continued to report pain, a second opioid analgesic, pethidine, was given. PCA with morphine was started immediately after patients were able to control the PCA pump in the PACU.

### Evaluation of chronic postsurgical pain

The incidence of the postoperative residual pain in the pelvis and at the scar area was evaluated in all 102 patients three months after surgery (according to the International Association for the Study of Pain chronic pain definition [29]), by a research nurse via telephone interviews. The following questions were asked to define CPP location and duration as well as painkiller consumption, as described elsewhere [30]: 1) Do you feel any pain at the scar area or pelvis?; 2) What is the duration of the pain?; and 3) Do you take any medication to alleviate the pain? If pain was reported, the NRS (0-no pain, 10-worst pain) was used to assess pain intensity. If patients reported an increase in pain over the past month or change in pain sensation, they were advised to contact the pain clinic. These patients were evaluated at the pain clinic using the DN4 Questionnaire, a screening tool for neuropathic pain [31,32].

### Genotyping

Whole blood samples were transported to the Department of Gene Technology at the Tallinn University of Technology, where genotyping analysis was performed [12]. Lymphocytes were isolated from blood specimens using Ficoll-Paque gradients, and genomic DNA was isolated using the Puregene DNA Purification Kit (Gentra Systems, Minneapolis, MN). Variants of the OPRM1 gene were prepared by DNA sequence analysis of polymerase chain reaction (PCR)-amplified DNA, using primers located in flanking intron sequence. *OPRM1* exon 1 (from the genomic sequence accession number NC000006 nucleotides 154402000–2800) was amplified by PCR with primers F1: CAGAAGAGTGCCCAGTGAAGA and R755: TACCTCCCCTCTTTCATCCTC and sequenced directly with the additional primer F177: CGCAGAGGAGAATGTCAGATG. *COMT* exon 4 (from the genomic sequence accession number NC000022, nucleotides 18331000–1400 SNP G>A, Val158Met) was amplified by PCR with primers F206: CTCATCACCATCGAGATCAAC and R301: CCTTTTTCCAGGTCTGACAAC. Amplified DNA was cut with the restriction enzyme Hin1 II. When Guanosine was added after gel agarose (2%) electrophoresis, 87 and 29 base pair long fragments were created. Adenosine gave rise to 69, 18, and 29 base pair long fragments. The genotyping error rate was directly determined by re-genotyping 25% of the samples randomly chosen for each locus. The overall error rate was determined to be < 0.005.

### Statistical analysis

Numerical variables were summarized using the mean and standard deviation, and categorical variables were summarized as N and percentage (%). Patients’ demographics and baseline characteristics between CPP and non-CPP patients were compared using t-test or Fisher’s exact test.

Before genetic association analysis, Hardy–Weinberg equilibrium (HWE) was first assessed via exact test and confirmed for the *OPRM1* and *COMT* SNPs. First, multiple logistic regression analysis was used to test all patients for association between CPP status and each SNP, adjusting for age, sex, and baseline pain score. We evaluated additive, dominant, and recessive genetic inheritance (i.e., gene-dosage effect) models for each SNP in relationship to CPP status, in which the genotypes were coded as (0, 1, 2), (0, 1, 1), and (0, 0, 1) based on the numbers of variant alleles. In other words, the homozygote of the wide-type or major allele was always used as the referent category for statistical comparison. Second, for the CPP patients, the association of each SNP with the intensity of CPP score (or duration of pain) was evaluated assuming three gene-dosage effect models after adjusting for sex, age, baseline pain score, and pain medication using the analysis of covariance (ANCOVA) method. Similar analyses were also performed for combined *COMT/OPRM1* genotypes.

All statistical analyses were performed using the SAS software, version 9.2 (SAS Institute, Cary, NC) and the PLINK software [33]. A *P*-value of less than 0.05 was considered to be statistically significant.

## Abbreviations

CPP: Chronic postsurgical pain; SNP: Single nucleotide polymorphism; COMT: Catechol-O-methyl transferase; OPRM1: Opioid receptor μ-1; CNS: Central nervous system; PACU: Post-anesthesia care unit; NRS: Numeric rating score; ASA: American society of anesthesiologists.

## Competing interests

We declare no conflict of the interests.

## Authors’ contributions

YK - Protocol design, protocol submission and management, data analysis, manuscript preparation. BG - Study conduction, data collection, manuscript drafting and review. AL - Study conduction, data collection, manuscript drafting and review. AV– Genetic study conduction, data collection, manuscript drafting and review. LQ – Data interpretation, manuscript review. FD – Statistical analysis, data interpretation, manuscript review. IB - Statistical analysis, data interpretation, manuscript review, editing and submission. All authors read and approved the final manuscript.

## Supplementary Material

Additional file 1: Table S1 Pain scores and duration of pain in 35 CPP patients stratified by gender. Click here for file

Additional file 2: Table S2 Distribution of genotypes by gender for *COMT* rs4680 and *OPRM1* rs1799971. Click here for file
