# Supplementary material for: Chronic pain after lower abdominal surgery: do catechol-O-methyl transferase/opioid receptor μ-1 polymorphisms contribute?
Source: Mol Pain. 2013 Apr 8;9:19. doi: 10.1186/1744-8069-9-19 (PMC3623849; doi:10.1186/1744-8069-9-19)
Supplement: Additional file 1: Table S1 — Pain scores and duration of pain in 35 CPP patients stratified by gender. [file 1744-8069-9-19-S1.doc]

**Supplemental Table 1**. Pain scores and duration of pain in 35 CPP patients stratified by gender

|  | **Male**  **(n = 14)** | **Female**  **(n = 21)** | ***P*-value** |
| --- | --- | --- | --- |
| Pain day 0 | 2.4 ± 1.3 | 2.5 ± 1.3 | 0.71 |
| Pain day 1 | 1.5 ± 1.0 | 1.8 ± 1.1 | 0.11 |
| Pain day 2 | 1.1 ± 1.0 | 1.1 ± 1.0 | 0.97 |
| CPP score | 3.2 ± 1.2 | 4.1 ± 1.6 | 0.09 |
| Duration of pain | 12.9 ± 16.5 | 8.6 ± 7.9 | 0.37 |

Note: *P*-value for Student’s *t*-test was shown.
