# Supplementary material for: Chronic pain after lower abdominal surgery: do catechol-O-methyl transferase/opioid receptor μ-1 polymorphisms contribute?
Source: Mol Pain. 2013 Apr 8;9:19. doi: 10.1186/1744-8069-9-19 (PMC3623849; doi:10.1186/1744-8069-9-19)
Supplement: Additional file 2: Table S2 — Distribution of genotypes by gender for COMT rs4680 and OPRM1 rs1799971. [file 1744-8069-9-19-S2.doc]

**Supplemental Table 2**. Distribution of genotypes by gender for *COMT* rs4680 and *OPRM1* rs1799971

|  | **Genotype** | **Male**  **(n = 45)** | **Female**  **(n = 57)** |
| --- | --- | --- | --- |
| rs4680 | AA | 10 | 12 |
|  | GA | 25 | 30 |
|  | GG | 10 | 15 |
| rs1799971 | GG | 2 | 1 |
|  | AG | 8 | 7 |
|  | AA | 35 | 49 |

Note: Cochran-Armitage trend test *P*-value for rs4680, rs1799971 was 0.70 and 0.25.
